# Expanding the TDP-43 Proteinopathy Pathway From Neurons to Muscle: Physiological and Pathophysiological Functions

**DOI:** 10.3389/fnins.2022.815765

**Published:** 2022-02-03

**Authors:** Lauren Versluys, Pedro Ervilha Pereira, Nika Schuermans, Boel De Paepe, Jan L. De Bleecker, Elke Bogaert, Bart Dermaut

**Affiliations:** ^1^Department Biomolecular Medicine, Faculty of Medicine and Health Sciences, Ghent University, Ghent, Belgium; ^2^Center for Medical Genetics, Ghent University Hospital, Ghent, Belgium; ^3^Department of Neurology and Neuromuscular Reference Center, Ghent University Hospital, Ghent, Belgium; ^4^Department of Head and Skin, Faculty of Medicine and Health Sciences, Ghent University, Ghent, Belgium

**Keywords:** TDP-43 proteinopathy, neurons, muscles, neurodegeneration, myopathy, ALS, FTD, FTLD

## Abstract

TAR DNA-binding protein 43, mostly referred to as TDP-43 (encoded by the *TARDBP* gene) is strongly linked to the pathogenesis of amyotrophic lateral sclerosis (ALS) and frontotemporal dementia (FTD). From the identification of TDP-43 positive aggregates in the brains and spinal cords of ALS/FTD patients, to a genetic link between *TARBDP* mutations and the development of TDP-43 pathology in ALS, there is strong evidence indicating that TDP-43 plays a pivotal role in the process of neuronal degeneration. What this role is, however, remains to be determined with evidence ranging from gain of toxic properties through the formation of cytotoxic aggregates, to an inability to perform its normal functions due to nuclear depletion. To add to an already complex subject, recent studies highlight a role for TDP-43 in muscle physiology and disease. We here review the biophysical, biochemical, cellular and tissue-specific properties of TDP-43 in the context of neurodegeneration and have a look at the nascent stream of evidence that positions TDP-43 in a myogenic context. By integrating the neurogenic and myogenic pathological roles of TDP-43 we provide a more comprehensive and encompassing view of the role and mechanisms associated with TDP-43 across the various cell types of the motor system, all the way from brain to limbs.

## Introduction

**FIGURE 1:**
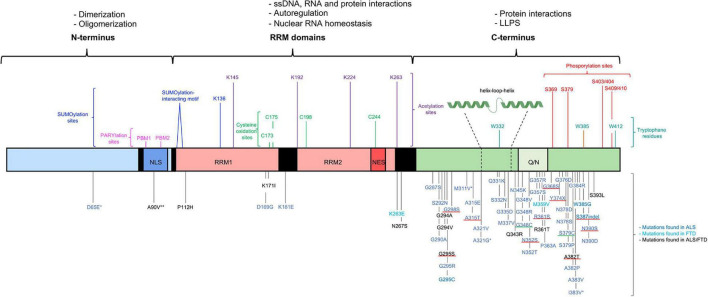
Structural representation of the structure of TDP-43, functions and mutations. TDP-43 contains three functional domains: the NTD (a.a. 1–102), two RRMs (a.a. 104–262) and the CTD (a.a. 274–414). The functional domains with the associated physiological functions on top of the figure. The most important residues associated with post-translation modifications and LLPS shown above the TDP-43 structure. More than 50 mutations are described and shown below the TDP-43 structure. Most of the disease-causing mutations are located in the CTD. The * benign mutations, ** potential risk-increasing variant. The disease-causing mutations inserting or deleting a phosphorylation residue with a well-known effect are underlined in red and the disease-causing mutations inserting a cysteine residue are underlined in green (Pesiridis et al., 2009; Millecamps et al., 2010; Solski et al., 2012; Van Blitterswijk et al., 2012; Chen et al., 2019, 2021; Agrawal et al., 2021; Klim et al., 2021; Tziortzouda et al., 2021).

A broad spectrum of neurodegenerative conditions belongs to the class of TDP-43 proteinopathies. In all these conditions, TDP-43 is the main protein found in inclusions in the brain therefore linking all these diseases to this common pathological category. TDP-43 proteinopathy is found in ALS, FTD, Alzheimer’s disease (AD), and limbic-predominant age-related TDP-43 encephalopathy (LATE) (Nelson et al., 2019; Prasad et al., 2019). TDP-43 proteinopathy is best established in the context of ALS and FTD. Numerous pathogenic mutations have been reported in the *TARDBP* gene, establishing the causal involvement of this protein in disease pathogenesis. The vast majority of the ALS-causing mutations are missense mutations located in the C-terminal region (Figure 1). ALS is a neuromuscular disorder characterized by progressive degeneration and loss of both the upper and lower motor neurons in the spinal cord and brainstem. Patients gradually develop symptoms going from muscle weakness and muscular atrophy to paralysis. These motor impairments progress rapidly and usually patients die within 3–5 years after the first disease symptoms (Prasad et al., 2019). ALS is one of the most common types of motor neuron diseases with a mean incidence of 5.4/100,000 in Europe. The disease usually starts in late adulthood, but in rare cases, juvenile (before 25y) and young-onset ALS (before 45y) can occur. 10% of ALS cases are familial (fALS), whereas in the largest subset of patients a clear family history is lacking and the disease takes a sporadic nature (sALS). Although mutations in the *TARDBP* gene are only present in 3% of fALS cases and 1.5% of sALS cases, neuropathological TDP-43 aggregates are noted in approximately 97% of the sALS cases (Grad et al., 2017; Prasad et al., 2019). Clinically, TDP-43 proteinopathies not only cause impairment of the motor system but are also related to cognitive deterioration. In FTD, a heterogeneous group of dementias, there is degeneration of the neurons in the frontal and temporal lobes. At the microscopic level, gliosis and microvacuoles are present in combination with synaptic and neuronal loss (Younes and Miller, 2020). The clinical manifestations are variable between patients and include social and personality changes as well as cognitive and language impairments (Prasad et al., 2019). In 40–50% of all FTD cases pathological TDP-43 inclusions are present, however, mutations in TDP-43 associated with FTD are extremely rare. Based on the morphology of TDP-43 inclusions, TDP-43-associated FTD is divided into four subtypes. In type A the cortical layers are affected by small intracytoplasmic TDP-43 inclusions and intranuclear TDP-43 inclusions are found in superficial cortical layers II and III. Type B is characterized by round neuronal TDP-43 inclusions especially in the cortex, type C with long TDP-43 immunoreactive neurites, and in type D neuronal intranuclear inclusions and neuronal cytoplasmic inclusions are observed. Together these diseases belong to a spectrum of neurodegenerative diseases representing the second most prevalent form of dementia (Younes and Miller, 2020). In the most common type of dementia being AD, amyloid-β plaques and neurofibrillary tangles stand as the major pathological hallmarks. Besides those, TDP-43 accumulations are observed in 20–50% of AD patients and remarkably in 75% of the patients suffering from severe AD (Jo et al., 2020). Recently, a new neurodegenerative disorder, LATE-neuropathological change (LATE-NC), has been described. Typically, at the pathological level there is accumulation of TDP-43 in limbic structures that may coexist with hippocampal sclerosis. Patients usually have, similar to AD, an amnestic form of dementia. Frequently, there is comorbidity of these two diseases. LATE manifests in older people, with the autopsy of *post-mortem* brains revealing the presence of LATE-NC in 20–50% of patients older than 80 years, further emphasizing a primary role for TDP-43 in neurodegeneration (Nelson et al., 2019).

As previously described, mutations in the *TARDBP* gene are mainly associated with ALS but these mutations only contribute to pathology in a small fraction of all ALS cases. In FTD and AD, mutations in the *TARDBP* gene are rare although the TDP-43 pathology is present in a quite large proportion of all cases (Brouwers et al., 2010; Prasad et al., 2019; Jo et al., 2020). This indicates that in most patients TDP-43 proteinopathy can manifest in the absence of *TARDBP* gene mutations, and that other genes are involved in TDP-43 proteinopathy. Indeed, several other genes are associated with TDP-43 proteinopathies as well, with the most important ones being *C9ORF72* in the ALS-FTD spectrum and *VCP* (valosin containing protein), *hnRNPA1 and hnRNPA2B* in multisystem proteinopathies (MSP) (Gitcho et al., 2009b; Kim et al., 2013; Taylor, 2015; Prasad et al., 2019; Jo et al., 2020). Despite the fact that several causal genes can be linked to TDP-43 pathology, the pathogenetic mechanism from gene defect to inclusion formation and disease onset is not fully understood. Interestingly, recent evidence is accumulating that indicates the spectrum of TDP-43 proteinopathy encompasses not only neurodegenerative conditions, but also myopathies. The first evidence for a role of TDP-43 in muscle tissues stems from the observation of TDP-43 aggregates in myopathies with rimmed vacuoles such as sporadic inclusion body myositis and *VCP* multisystem proteinopathy-associated inclusion body myopathy (Küsters et al., 2009; Olivé et al., 2009). Over the past few years, a growing body of evidence has been gathered pointing toward an up until now underappreciated but seemingly important role for TDP-43 in muscle physiology and disease (Militello et al., 2018; Vogler et al., 2018).

The aim of this review is to provide an up-to-date overview of the many different physiological and pathophysiological roles of TDP-43, with a special focus on its tissue-specific functions. Furthermore, we will highlight the role of TDP-43 in muscle tissue and the current findings relating to this less well-studied facet of TDP-43 proteinopathies.

## Structure of TDP-43

**FIGURE 2:**
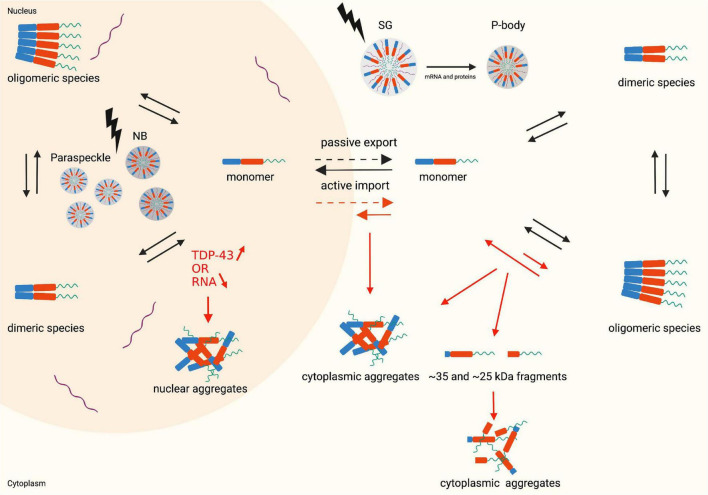
Monomer, dimer and oligomer equilibrium state, the nucleocytoplasmic shuttle of TDP-43 in physiological (black arrows) and pathophysiological (red arrows) conditions and structural representation of LLPS (lightning bolt). Nuclear aggregates are formed when the TDP-43 levels in the nucleus increase, or the RNA levels decrease. An altered monomer-oligomer equilibrium causes the formation of cytoplasmic aggregates and cleaved C-terminal fragments which subsequently form aggregates. During stress conditions the cells undergo LLPS resulting in the formation of membraneless organelles: nuclear bodies and paraspeckles in the nucleus and stress granules and P-bodies in the cytoplasm. Created with BioRender.com.

TDP-43 is a nuclear 414 amino acid RNA/DNA binding protein encoded by the human *TARDBP* gene (Prasad et al., 2019). The *TARDBP* gene is located at chromosome 1p36.22 and is made up of 6 exons (Wang et al., 2004). TDP-43 is a ubiquitously expressed protein which is highly conserved across different species (Prasad et al., 2019). TDP-43 was initially identified in 1995 as a transcriptional repressor of HIV-1 (Ou et al., 1995). Nowadays it is clear that TDP-43 belongs to the heterogeneous nuclear ribonucleoprotein (hnRNP) family, which are mainly nuclear proteins well-known for their nucleic acid and protein binding capacities. Members of this family participate in a variety of processes related to the nucleic acid metabolism such as processing and modification of RNA, transcription, translation, stabilization, and transport of RNA (Han et al., 2010). Comprehending the structure of TDP-43 is a key step to understand how this protein is capable to act in such a broad range of processes and hence explain its diverse cellular functions. Three key domain types can be distinguished: an N-terminal domain (NTD), two RNA-recognition motifs (RRMs) and a C-terminal domain (CTD) (Figure 1; Prasad et al., 2019). All domains are regulated by post-translational modifications to finetune their functions. The NTD plays a crucial role in determining the type of TDP-43 species present in the cell: monomers, dimers or oligomers. The dimeric and oligomeric forms of TDP-43 are thought to be both functionally active species (Zhang et al., 2013; Afroz et al., 2017; Jiang et al., 2017). An equilibrium between the monomeric and dimeric species and between monomeric and oligomeric species exists and it is speculated that these equilibriums are one of the many ways of monitoring the activity of TDP-43 in a cell (Figure 2; Shiina et al., 2010; Afroz et al., 2017). How monomeric species transform to dimers or oligomers and what the precise triggers are for these processes is not fully understood, however, it is definitely known that the NTD plays a crucial role in these processes (Zhang et al., 2013; Afroz et al., 2017). Two types of TDP-43 dimers have been described. One dimer species is formed by N-terminal head-to-head interactions, whereas the other dimer originates from N-terminal head-to-tail interactions (Wang Y.T. et al., 2013; Wang et al., 2018). Both dimers, are stabilized by residues participating in the interface. Hereto hydrophobic residues and ß-strands/sheets are formed which are further strengthened by tertiary interactions or self-interactions in the RRMs (Chang et al., 2012; Zhang et al., 2013; Mompeán et al., 2017). In oligomers a head-to-tail interaction of the NTD is suggested. This head-to-tail interaction is established by charge complementation and is reinforced by salt bridges, hydrophobic contacts and hydrogen bounds (Afroz et al., 2017). Also, in this conformation, stabilization by interface residues is essential and the formation of these oligomers may occur in a concentration-dependent manner (Chang et al., 2012). Correct functioning of the NTD-driven dimerization and oligomerization is therefore considered crucial for physiological TDP-43 functioning.

Both RRMs are mainly involved in the binding to a pleiotropy of interaction partners and display a high affinity to ssDNA, RNA and proteins. The association between nuclear RNA and the RRMs is important to retain the protein in the nucleus. This is a concentration-dependent process and allows control of cytoplasmic TDP-43 levels by regulation of the free TDP-43 available for passive diffusion to the cytoplasm (Duan et al., 2021). Hence, steady-state cytoplasmic TDP-43 levels are dependent on the RNA concentration in the nucleus. Both RRMs are involved in the binding of RNA, but RRM1 determines the affinity of TDP-43 for GU-rich sequences which are mainly present in intronic pre-mRNAs, but also in the 3′ UTR and some exons (Ayala et al., 2011; Polymenidou et al., 2011; Vogler et al., 2018). The second RRM, RRM2, shows no sequence preference but increases the binding affinity for RNA (Ayala et al., 2011). Besides the synergistic effect on the RNA binding, the high evolutionary conservation of the RRM2 is indicative of a more specific but still unknown function. Apart from a general RNA binding function of the RRM1, this domain plays a key role in the autoregulatory process of TDP-43. The TDP-43 protein controls its own expression levels through binding to its own mRNA. The RRM1 of the TDP-43 protein binds the pre-mRNA at the TDP-43 binding region (TDPBR) located in in the 3′UTR region (Buratti and Baralle, 2011; Prasad et al., 2019). The TDPBR contains low-affinity binding sites for TDP-43 suggested to be advantageous for the regulatory process as numerous TDP-43 molecules will be required to trigger a response (Ayala et al., 2011). Under normal circumstances a transcript that uses the most efficient polyadenylation site pA1 is responsible for the majority of the TDP-43 protein production. However, when the TDP-43 protein concentration increases a higher number of TDP-43 proteins binds to the TDPBR of the nuclear pre-mRNA leading to alternative splicing and the incorporation of an intron which include the TDBPR and pA1. This creates a novel transcript with suboptimal polyadenylation sites and the formation of unstable mRNA. As a consequence, part of the TDP-43 mRNA is retained longer in the nucleus where it degrades rapidly (Ayala et al., 2011; Pons et al., 2017). TDP-43 levels are therefore tightly controlled by its own protein levels; a process mainly regulated by the RRM1 domain, but speculated to be finetuned by the recruitment of other proteins through a region in the CTD (Ayala et al., 2011).

The CTD is an intrinsically disordered region with a low sequence complexity resembling a prion-like domain (PLD), containing glycine and uncharged polar amino acids (a.a.). This PLD is an important factor in regulating many protein-protein interactions and the liquid-liquid phase separation (LLPS) behavior of TDP-43 (Prasad et al., 2019). Although the CTD is able to induce phase separation on its own, the NTD induced oligomerization can enhance this physical process (Wang et al., 2018).

## Subcellular Localization of TDP-43

Mislocalization of TDP-43 in the cytoplasm is typically observed in pathological conditions, hence understanding the regulation of its normal subcompartmentalization is of great importance. TDP-43 is mainly localized in the nucleus but continuously shuttles to and from the cytoplasm (Figure 2). Part of this nucleocytoplasmic transport of TDP-43 is mediated by carrier proteins, karyopherins, which function as importins or exportins. Nuclear import of TDP-43 is an active process mediated by karyopherin-α (KPNA) and karyopherin-β1 (KPNB1). KPNB1 is able to bind KPNA after it recognizes the nuclear localization signal (NLS) of TDP-43, located in the NTD. This trimeric complex is transported through the nuclear pore into the nucleus. In the nucleus, the Ras-related nuclear protein guanosine triphosphate (Ran-GTP) binds KPNB1 resulting in a dissociation of this complex and the release of TDP-43. The remaining complex components, KPNB1 and Ran-GTP, are immediately transported back to the cytoplasm, in contrast to KPNA which requires binding to the nuclear export factor Chromosome Segregation 1 Like for its translocation to the cytoplasm. This latter transport is also dependent on Ran-GTP binding as an energy source. Back in the cytoplasm, hydrolysis of GTP to GDP causes the dissociation of the complexes (Nishimura et al., 2010; Mihevc et al., 2017). Upon import, TDP-43 binds nuclear RNA and is in this way retained in the nucleus (Duan et al., 2021). Despite the well-described nuclear import pathway of TDP-43, the routes for nuclear export are still debated. Some reports suggest an active transport of TDP-43 to the cytoplasm which depends on exportin-1 (XPO). In the nucleus, XPO1 binds the nuclear export signal (NES) located in RRM2 of TDP-43 together with Ran-GTP. This complex is then translocated to the cytoplasm, where the hydrolysis of GTP to GDP results in the dissociation and hence the cytoplasmic release of TDP-43 (Mihevc et al., 2017). However, replacement of the XPO1 binding residues in TDP-43 by alanine does not disrupt the nuclear export of TDP-43 in HeLa cells (Ederle et al., 2018). Likewise, a complete deletion of the NES by removing the entire RRM2 does not alter the subcellular localization of TDP-43, suggesting that the nuclear export is independent of the RRM2 in which the NES is located (Pinarbasi et al., 2018). Although there is an increase, albeit limited, in cytoplasmic TDP-43 upon overexpressing of XPO1, inhibition of XPO1 using SINE compounds does not alter the localization of TDP-43 in primary rat cortical neurons (Archbold et al., 2018). Furthermore, the binding affinity of the NES of TDP-43 for XPO1 is weak and both in HeLa cells and rat hippocampal neurons XPO1 is proven not to be essential for TDP-43 localization (Pinarbasi et al., 2018). A second route of active transportation that has been suggested in the literature occurs via the mRNA export pathway which involves the heteromeric TAP/p15 export receptor and the TREX complex (Ederle and Dormann, 2017). Silencing of Aly/REF, a key factor of the mRNA transportation machinery, however, still results in the export of TDP-43, indicating that TDP-43 nuclear export is not primarily dependent on the mRNA export machinery (Ederle et al., 2018). A third active transportation route via exportin-5 (XPO5) was also suggested but silencing of XPO5 in HeLa cells and its overexpression in rodent primary neurons shows that XPO5 is not required for the export of TDP-43 (Archbold et al., 2018; Ederle et al., 2018). Altogether these data suggest that nuclear export of TDP-43 is independent of active transport but rather occurs via passive diffusion. This idea is further supported by the observed nuclear retention when TDP-43 is artificially enlarged, thereby strengthening the passive diffusion hypothesis as a size-dependent process (Ederle et al., 2018; Pinarbasi et al., 2018).

All in all, the proper regulation of the nucleocytoplasmic trafficking is important for allowing TDP-43 to exert its proper physiological functions via active re-uptake of TDP-43 protein to the nucleus, but also to maintain steady-state levels.

## Physiological Functions of TDP-43

The specific functions of TDP-43 are mainly studied in the central nervous system (CNS) as TDP-43 is a common pathological hallmark in patients suffering from ALS, FTD, and other neurodegenerative diseases. As can be expected, taking into account the complexity of the protein structure, the different protein species, and the variable subcellular localization; TDP-43 is a protein that participates in many cellular and molecular processes.

At a cellular level, TDP-43 plays an important role during embryonic and post-natal development in general and more specifically it has crucial functions in the development and maintenance of the nervous system. TDP-43 is continuously expressed at high levels during embryonic development, followed by a gradual decline during postnatal development. TDP-43 knock-out mice die during the early embryonic stage, knock-down in zebrafish is associated with motor deficits, and depletion of the *Drosophila melanogaster* (*D. melanogaster*) homolog, referred to as *TBPH*, results in reduced eclosion and shorter lifespan (Kabashi et al., 2009; Sephton et al., 2010; Vanden Broeck et al., 2013). Loss of the *C. elegans* homolog produces contradictory results. While some studies report that loss of the TDP-43 homolog results in reduced growth, fertility and locomotion, others report an increase in lifespan and enhanced susceptibility to stress due to changes in the insulin/IGF pathway (Vaccaro et al., 2012; Zhang et al., 2012; Vanden Broeck et al., 2014). During the postnatal stage, postpartum knock-out of TDP-43 in mice results in loss of body fat followed by a quick death, whereas a selective elimination of TDP-43 in the postnatal motor neurons of mice causes morphological changes in the motor neuron system resulting in motor dysfunction (Chiang et al., 2010; Iguchi et al., 2013). These data indicate that TDP-43 is crucial for development, survival and might play a role in metabolic processes.

At a molecular level, TDP-43 behaves like other members of the hnRNP family involved in the regulation of gene expression. TDP-43 exerts this function by acting in multi-protein complexes interacting with many different targets involved in the different levels of gene regulation ranging from transcription to RNA transport and translation. These targets consist of both nucleic acids (RNA, DNA) and proteins (Han et al., 2010). The fact that TDP-43 is a promiscuous binding protein explains the ability of the RRMs to interact with so many different targets, which is reflected by the more than 6000 RNA binding targets of TDP-43 (Polymenidou et al., 2011). This promiscuity is explained by the nature of these interactions, which are numerous but mostly limited to weak interactions. Also, when it comes to binding proteins, TDP-43 is not selective. As mentioned previously, the C-terminal region of TDP-43 harbors an intrinsically disordered protein region (IDPR). IDPRs are promiscuous binders, known to interact in various protein-protein and protein-nucleic acid interactions through highly specific but weak interactions (Uversky, 2013). Beside the involvement in RNA regulation, a second important group of TDP-43 functions is attributed to the fact that TDP-43 can contribute to the formation of membraneless organelles via its LLPS capacity, mediated by the CTD (Prasad et al., 2019). This is, likewise, based on the promiscuity of the interactions, again highlighting the extended binding capacity of TDP-43 and its importance for proper cellular functioning.

### RNA Maturation, Stabilization, Transport, and Translation

A well-described function of TDP-43 is its regulatory role in RNA splicing and this through either direct interaction with the RNA itself, or interaction with proteins involved in the splicing machinery (Freibaum et al., 2010). In general, TDP-43 regulates the splicing patterns of numerous RNA transcripts by binding UG-repeats near the splice sites of pre-mRNA (Tollervey et al., 2011). Besides the involvement in the normal splicing process, TDP-43 is suggested to have a role in alternative splicing events as well. This role for TDP-43 is especially reported for two specialized forms of splicing, cryptic exon splicing and splicing of cassette exons (Polymenidou et al., 2011; Ling et al., 2015). Cryptic exons are flanked by long UG-repeats and are therefore recognized by TDP-43. Upon interaction, TDP-43 suppresses the incorporation of these exons in the mRNA transcript. Cryptic exons often contain premature stop codons leading to nonsense-mediated decay. However, in physiological conditions nuclear TDP-43 prevents the usage of these exons (Ling et al., 2015). Additionally, a large number of cassette exons are also regulated by TDP-43, and by doing so, TDP-43 mediates alternative splicing of transcripts (Polymenidou et al., 2011).

When looking at splicing, tissue-specific functions for TDP-43 emerge, namely between neuronal and muscular tissues. In neurons, TDP-43 regulates the splicing patterns of transcripts encoding proteins involved in neuronal processes and the number of splicing events is higher when compared to muscle cells (Šušnjar et al., 2021; Tollervey et al., 2011). The repertory of RNA binding proteins (RBPs) present in neurons is different from that in muscle cells, which also exhibit relatively higher expression levels. The cooperation between TDP-43 and these RBPs contributes to a complex splicing regulation, especially in neurons, explaining the higher prevalence of TDP-43 regulated splicing in neurons (Šušnjar et al., 2021). In contrast, muscular TDP-43 modulates splicing patterns of mRNAs encoding proteins involved in DNA-related processes, which are important in muscle differentiation. Furthermore, TDP-43 targets mRNAs of proteins involved in striated muscle development and muscle differentiation (Šušnjar et al., 2021). In *D. melanogaster, TBPH* is responsible for the regulation of the pre- and postsynaptic Discs large protein (Dlg) levels in muscles and motoneurons by binding the Dlg mRNA and in this way regulating its splicing patterns. Of note, Dlg has an important role in the formation of neuromuscular junctions and organization of the glutamate receptors on muscle membranes, suggesting an important role for *TBPH* in muscle tissues (Strah et al., 2020).

Besides splicing, TDP-43 controls the biogenesis of non-coding RNAs, such as micro-RNA (miRNA), small nucleolar RNA (snoRNA) and long non-coding RNAs (lncRNA). The regulation of miRNAs occurs either through direct binding of TDP-43 to the primary miRNAs in the nucleus and their precursors in the cytoplasm, or by a protein-protein interaction between TDP-43 and the nuclear Drosha and cytoplasmic Dicer complexes. The association between TDP-43 and the Drosha or Dicer complexes is mediated by several RNA species and the CTD (Kawahara and Mieda-Sato, 2012). The putative role for TDP-43 in snoRNA processing comes from experiments in *D. Melanogaster* as loss-of-function (LOF) and gain-of-function (GOF) of *TBPH* results in high upregulation of snoRNAs (Vanden Broeck et al., 2013). In the human brain, TDP-43 binds lncRNAs, transcripts with more than 200 nucleotides enriched in UG-repeats. Well-known lncRNAs regulated by TDP-43 are nuclear paraspeckle assembly transcript 1 (*NEAT1*) and metastasis associated lung adenocarcinoma transcript 1 (*MALAT1*). *NEAT1* is involved in the structural formation of paraspeckles, whereas *MALAT1* acts as a splicing regulator by modulating the distribution of splicing factors in paraspeckles and the phosphorylation of splicing factors (Polymenidou et al., 2011; Tollervey et al., 2011). In muscle, an interaction between TDP-43 and miRNAs or lncRNAs has been described as well. Based on *in vitro* and *in vivo* experiments TDP-43 interacts with the microRNA-1 (miR-1) family that promotes differentiation of striated muscle progenitors. TDP-43 negatively regulates miR-1 activity by limiting its interaction with the RNA-induced silencing complex which is required for miRNA to perform its activity. Overexpression of human TDP-43 in mice models decreases the activity of the miR-1 family which in turn increases the translation of the miR-1 targets, IGF-1 and HDAC4. HDAC4 is associated with muscle denervation and blocks innervation. This indicates that in steady-state conditions the miR-1 targets are suppressed, therefore the interaction between TDP-43 and miR-1 is required to maintain homeostasis of the miR-1 targets, and subsequently mediate muscle development and preserve balance in adult muscle tissues (King et al., 2014). Furthermore TDP-43 interacts with *Myolinc*, a muscular-specific lncRNA. This interaction is important for the binding of TDP-43 to the promotor of *MyoD* and other myogenic commitment regulatory factors. This interaction is essential for the expression of muscle-specific genes, emphasizing the crucial role of TDP-43 in the differentiation from myoblasts to myotubes (Militello et al., 2018).

Although it has been previously described that TDP-43 is mainly localized in the nucleus, even in steady-state conditions a small proportion of TDP-43 is located in the cytoplasm. This cytoplasmic TDP-43 binds to the 3′-UTR of several mature mRNAs, thereby participating in the regulation of their stability, transport and translation (Polymenidou et al., 2011; Tollervey et al., 2011). Transport and translation are controlled by binding to numerous proteins as well (Freibaum et al., 2010). TDP-43 interacts with RBPs to transport the mRNAs to their target location as messenger ribonucleoproteins (mRNP) (Alami et al., 2014). Proteomic studies reveal that TDP-43 associates with elongation factors and proteins involved in translation initiation (Freibaum et al., 2010). On top of that, excess of cytoplasmic TDP-43 represses translation by binding to Receptor for activated C kinase 1 in order to modulate the activity of the translation initiation binding protein and the translation initiation factor, 4E-BP1 and eIF4E (Russo et al., 2017). In neuronal cells TDP-43 is responsible for the maintenance and stability of mRNAs encoding hNFL, choline acetyltransferase, progranulin and mRNAs involved in synaptic activity, defining the tissue-specific relevance (Strong et al., 2007). These mRNAs are transported as mRNP granules along dendrites and axons through a tightly regulated process, important for neuronal development and synaptic plasticity. Cooperation between TDP-43 and other RBPs is responsible for the transport of these mRNP granules and facilitates the transfer of the mRNAs to their destination (Alami et al., 2014). There are two well-known RBPs that associate with TDP-43 in the transport process, fragile X mental retardation protein (FMRP) and Staufen 1. FMRP regulates the anterograde transport of dendritic mRNP granules, whereas Staufen 1 is involved in the retrograde transport of mRNP in neuronal dendrites (Chu et al., 2019). Strictly associated with mRNA transport, is the translation of mRNA. TDP-43 inhibits the translation by directly interacting with the elongation factors and other ribosomal proteins (Wang et al., 2008). Apart from the collaboration between TDP-43 and FMRP in mRNA transport, this interplay between these two proteins is important in translation as well. Cooperation between TDP-43 and FMRP causes translational repression of mRNAs lacking a G-quadruplex motif such as Rac1, GluR1, and Mapb1. TDP-43 binds the UG-motifs of the mRNAs and subsequently acts as an adaptor to recruit the repression complex FMRP-CYFIP1, which binds eIF4E. The eIF4E-FMRP-CYFIP1 complex is then able to inhibit the ribosome attachment to the 5’-cap site of the mRNAs and in this way inhibit translation at the initiation step (Majumder et al., 2016). Besides the well-described role of TDP-43 as a translational repressor, there is also evidence that TDP-43 can act as a translation enhancer of specific mRNAs such as *CAMTA1* and *DENND4A* (Neelagandan et al., 2019). This variety of translational targets and functions of TDP-43 further demonstrates the important role of TDP-43 in regulating neuronal cell function and identity.

### Liquid-Liquid Phase Separation

Liquid-liquid phase separation is a reversible thermodynamic process characterized by the de-mixing of two distinct liquid phases. This process is responsible for the formation of membraneless liquid-like droplets, in which the components are still able to diffuse into the surrounding nucleoplasm or cytoplasm (Shin and Brangwynne, 2017; Gomes and Shorter, 2019). These membraneless organelles usually consist of proteins and RNA molecules, referred to as ribonucleoprotein bodies, and are important in a pleiotropy of cellular functions (Shin and Brangwynne, 2017). LLPS is driven by transient intermolecular interactions underlying subcellular organization (Shin and Brangwynne, 2017; Sun and Chakrabartty, 2017). The driving force for the assembly of the liquid-like droplets are protein-protein and protein-nucleic acid interactions mediated by both the C- and N-terminal domains, and the RRMs, respectively (Shin and Brangwynne, 2017). The CTD of TDP-43 harbors a disordered PLD that is enriched in the uncharged polar amino acids, glutamine and asparagine (Prasad et al., 2019). Secondary structures, together with aromatic residues located within the PLD, play a crucial role in this phase separation process (Conicella et al., 2016; Li et al., 2018). A cooperatively formed α-helix (a.a.: 321–330) is present in 50% of cases and interacts with helices of other TDP-43 molecules (Figure 1). Together with the α-helix, conserved residues (a.a.: 331–340) form a helix-loop-helix enhancing its helicity and stability upon self-interaction (Conicella et al., 2016). In addition to the helix-loop-helix region, there are some aromatic residues located in the PLD, crucial for LLPS as well. The most important ones are three tryptophan (W) residues: W334, W385, and W412. Tryptophan residue 334, located in the helix-loop-helix, strongly enhances the propensity toward self-assembly of the helical element. All these intermolecular interaction mechanisms are important to facilitate LLPS and the formation of liquid-like droplets (Li et al., 2018). Plus, although the CTD on its own is sufficient enough to induce LLPS, NTD-induced oligomerization can further enhance phase separation (Wang et al., 2018). Finally, the heteromolecular protein-RNA interaction between the RRMs of TDP-43 and accumulating RNA contributes to the formation of liquid-like droplets. A balanced ratio between TDP-43 and RNA is required for the assembly of membraneless organelles and to maintain the organelles in a dynamic state preventing the formation of toxic TDP-43 species (Maharana et al., 2018; Mann et al., 2019). The data presented above strongly highlights the contribution and importance of all the TDP-43 domains in the formation of membraneless organelles.

Besides such protein and RNA interactions LLPS is modulated by post-translational modifications as well. First of all, the NTD of TDP-43 contains two Poly(ADP-Ribose) (PAR)-binding motifs (PBM), PBM1 and PBM2, which interact with the PAR biopolymer, a sugar covalently linked to proteins. This binding results in the initiation of LLPS and the formation of stress granules (McGurk et al., 2018). Secondly, also phosphorylation has been shown to modify the phase separation behavior of TDP-43, with phosphomimic mutants in the NTD and CTD showing reduced phase separation (Wang et al., 2018; Gruijs da Silva et al., 2021).

The membraneless organelles can originate via LLPS in either the nucleus or the cytoplasm, resulting in varied functions and properties (Figure 2). Therefore this topic merits a more detailed discussion, as outlined in the following subsections.

#### Nucleus

##### Nuclear Bodies

TDP-43 undergoes phase separation in response to various stressors into reversible nuclear granules with cytoprotective functions such as interrupting DNA transcription and RNA processing which can be resumed when the stress has passed.

These membraneless nuclear granules are defined as nuclear bodies (NBs) as they are visible with a microscope, collect specific nuclear factors and the components shuttle between the granule and the surrounding nucleoplasm. Cell-based studies indicate the relevant, but distinct roles, of the RRMs in the formation of these NBs. The different functions can probably be attributed to the interaction with diverse RNAs, since RRM1 suppresses LLPS by indiscriminate RNA binding and conversely is primarily involved in the formation of LLPS by binding *NEAT1* lncRNA, whereas RRM2 suppresses LLPS by binding tRNA *in vitro*. During stress conditions *NEAT1* lncRNA is upregulated as a defense mechanism against stress and pathological conditions in neurons (Wang et al., 2020).

##### Paraspeckles

The function of nuclear paraspeckles is not completely characterized yet, but it is hypothesized that it is involved in the regulation of gene expression of proteins or mRNAs with inverted repeats in their 3′UTR (Hirose et al., 2014; Imamura et al., 2014). The main agonist to the formation of paraspeckles is *NEAT1*, a long non-coding RNA with a strong binding affinity for TDP-43. *NEAT1* has a short and a long isoform, *NEAT1_1* and *NEAT1_2* respectively. Both transcript isoforms are elements in the structural integrity of paraspeckles, however, only *NEAT1_2* is essential in the formation of the paraspeckles (Shelkovnikova et al., 2018; Matsukawa et al., 2021). *NEAT1_2* consists of three domains. The middle domain in turn, contains three functional subdomains that are responsible for the recruitment of the NONO dimer triggering oligomerization with other paraspeckle proteins and leading to paraspeckle assembly (Yamazaki et al., 2018). Curiously, the *NEAT1_2* isoform is found to be upregulated in TDP-43 knock-down neuronal cell lines. Additional neuronal cell-based experiments suggest that upregulation of *NEAT1_2*, and hence paraspeckle formation, is protective against cell death in cells in which there is endogenous dsRNA accumulation or impaired miRNA biogenesis as a consequence of loss of TDP-43 function (Shelkovnikova et al., 2018). Besides the fact that TDP-43 interacts with *NEAT1*, it acts as a paraspeckle protein itself, being required for paraspeckle formation (Naganuma et al., 2012; Nishimoto et al., 2013). The above data indicate that the upregulation of *NEAT1* in abnormal conditions may provide protection against toxicity.

#### Cytoplasm

##### Stress Granules

Stress granules (SGs) are cytoplasmic membraneless organelles formed during stress conditions, e.g., oxidative stress, osmotic stress, temperature and pH changes. SGs contain mainly non-translating mRNAs and, translation and transcription factors (Decker and Parker, 2012; Lee et al., 2021). In these LLPS-driven granules, non-essential proteins form gel-like membraneless organelles in the cytoplasm that are dynamic liquid droplets, allowing for temporary and reversible capturing of transcripts that are unnecessary under stress conditions. In this way, these transcripts are protected from damage and breakdown, and once the stress has subsided the transcripts can be released and transported back to the nucleus through rapid dissociation of the granules and resume their functioning (Harrison and Shorter, 2017; Sun and Chakrabartty, 2017).

There are some conflicting opinions as to whether TDP-43 is a crucial component of these SGs. Several studies show the presence of endogenous TDP-43 in SGs (McDonald et al., 2011; Parker et al., 2012). Colombrita et al. indeed show the presence of TDP-43 in SGs, however, not as a crucial element, whereas in other studies there are indications that TDP-43 is required for the formation of SGs as depletion of TDP-43 affects the SG dynamics (Colombrita et al., 2009; McDonald et al., 2011; Khalfallah et al., 2018). These discrepancies in results might be explained by the cell type and the stressor used. In NSC34 cell-lines TDP-43 is recruited into SGs in response to oxidative and osmotic stress, whereas in HEK239T cell-lines there is only a recruitment of TDP-43 in response to osmotic stress (Dewey et al., 2011).

Interestingly, Wang et al. suggest the formation of NBs by the upregulation of *NEAT1* lncRNA which reduces the chance to form cytoplasmic SGs and therefore prevents a build-up of cytoplasmic TDP-43 (Wang et al., 2020).

##### Processing-Bodies

Processing bodies (P-bodies) are cytoplasmic mRNP granules consisting of non-translating mRNA, RNA-binding proteins and other proteins. P-bodies are present in physiological conditions but can be upregulated during stress conditions to regulate translation and mRNA degradation (Decker and Parker, 2012; Lee et al., 2021). It is known that P-bodies and SGs interact with each other and exchange mRNAs and proteins (Harrison and Shorter, 2017; Fernandes et al., 2020). The role of TDP-43 in P-bodies has not yet been extensively studied although colocalization between P-bodies and TDP-43 has been reported (Wang et al., 2008; Fernandes et al., 2020). A recent study shows that TDP-43 expressing cell-lines treated with arsenite (oxidative stress) and subsequently sorbitol (osmotic stress) results in excessive accumulation of TDP-43 in the cytoplasm. During the recovery period from this stress exposure TDP-43 may be sequestered into P-bodies to further process mRNAs (Lee et al., 2021).

##### Myo-Granules

In muscle tissue there is a specific counterpart of the stress granules, referred to as myo-granules. Skeletal muscles are required for movement and for the body’s structural integrity, but also have important endocrine and metabolic functions (Yin et al., 2013; Fealy et al., 2021). The muscle tissue is composed of myofibers which contain the functional units, known as sarcomeres, and houses muscle stem cells (MuSCs) (Yin et al., 2013; Wheeler et al., 2021). The MuSCs typically reside in a quiescent state but become activated after damage. The repair of myofibers is mainly driven by the activated MuSCs that progress through regulated myogenesis. The activated MuSCs proliferate and differentiate in order to rebuild the damaged muscle fibers. This process of myogenesis is complex and mediated by several genes. The role of different RNA-binding proteins in myogenesis is reported in the literature, including the role of TDP-43. TDP-43 is mainly present in the earlier stages of myogenesis, and the TDP-43 levels are found to be at their highest in Pax7-positive MuSCs and in regenerating myofibers 5 days after injury (Wheeler et al., 2021). During muscle regeneration TDP-43 re-localizes to the cytoplasm and exists as a component of amyloid-like myo-granules. TDP-43 in myo-granules has been described as binding preferentially to mRNAs encoding for important structural sarcomeric proteins. In this way, TDP-43 facilitates local regulation of these mRNAs, important during the first phases of regeneration (Vogler et al., 2018).

## Pathophysiological Role of TDP-43

The pathophysiological hallmarks and alterations in cellular processes contributing to TDP-43 proteinopathy are similar in patients with or without *TARDBP* mutations. The key hallmarks of TDP-43 proteinopathy are cytoplasmic TDP-43 accumulations, accompanied by a nuclear depletion as cytoplasmic TDP-43 “captures” nuclear TDP-43. Three possible hypotheses giving rise to TDP-43 proteinopathy are still debated: a gain-of-function – novel gain of toxic function – or loss-of-function (Vanden Broeck et al., 2014). From the pleiotropic functions of TDP-43 it is easy to understand that balanced levels of TDP-43 are required for the protein to function correctly. These levels are managed by a number of different mechanisms. Besides the autoregulatory process, other processes contribute to keep steady-state levels; nucleocytoplasmic transport, LLPS, mitochondrial function, autophagy and RNA binding all have been shown to be critical for TDP-43 homeostasis. In some pathological conditions these mechanisms are disturbed resulting in altered TDP-43 levels or an unbalance of the various TDP-43 species due to post-translational modifications. How TDP-43 proteinopathy arises in diseased conditions will be further discussed in the following subsections.

### Impairment of the Autoregulatory Process and Defects in Nucleocytoplasmic Shuttling

Since the autoregulatory feedback loop is one of the key mechanisms balancing the protein levels, dysfunction of this loop could easily contribute to TDP-43 pathology. Interestingly, mutations in the 3′UTR which might impair this autoregulatory mechanism have been described in FTD patients, indicating that tight control of TDP-43 levels is of crucial importance (Gitcho et al., 2009a). Moreover, in TDP-43 proteinopathy, cytoplasmic TDP-43 aggregates often coincide with a nuclear depletion of TDP-43 (Prasad et al., 2019). Consequently, due to lowered nuclear protein levels, the autoregulatory pathway responds by increasing protein production. This results in a vicious cycle as increased TDP-43 levels trigger the formation of larger aggregates, a higher level of cytoplasmic mislocalization and lead to more nuclear depletion, which in turn increases the production of TDP-43 once more (Baralle et al., 2013). Typically, in disease conditions, the subcellular distribution of TDP-43 is altered, although no clear disease-causing mutations have been found in the NES or NLS of TDP-43. This could be explained by embryonic lethality caused by mutations in these two regions. In fact, the majority of disease-linked variants are found within the CTD, a domain not directly involved in the nucleocytoplasmic shuttle, often giving rise to toxic cytoplasmic accumulation (Barmada et al., 2010; Prasad et al., 2019). Interestingly, a correlation between TDP-43 pathology and dysfunction of the nuclear pore complex exists. Cytoplasmic mutant TDP-43 aggregates trigger impairment of the nucleocytoplasmic transport by sequestration and mislocalization of nucleoporins, the main constituents of the nuclear pore complex (Chou et al., 2018). Due to this, the active import pathway of TDP-43 is impaired, leading to increased levels of TPD-43 in the cytoplasm. This impairment in nucleoporins is also observed in motor neurons derived from ALS patients and is accompanied by TDP-43 mislocalization (Aizawa et al., 2019). Using NLS deficient forms of TDP-43, the importance of a correctly functioning nucleocytoplasmic shuttle becomes evident. HEK-293T cells overexpressing NLS-mutated TDP-43 sequester endogenous insoluble TDP-43 into the cytoplasm. As a consequence, the cells exhibit nuclear depletion of TDP-43 and accumulation of cytoplasmic aggregates containing ubiquitinated and toxic C-terminal species, typical hallmarks of TDP-43 pathology (Winton et al., 2008). In transgenic mice the expression of the NLS-mutated variant of TDP-43 in the forebrain manifests as neuronal death, corticospinal tract degeneration resulting in spasticity and a decrease of endogenous mouse TDP-43. In contrast to studies in cell cultures, in transgenic mice almost no insoluble aggregates are observed. This indicates that aggregation alone is not sufficient to induce neuronal loss and that the loss of endogenous TDP-43 may contribute to neurodegeneration (Igaz et al., 2011).

The data detailed above highlight the importance of an intact autoregulatory pathway in combination with a well-functioning nucleocytoplasmic shuttling, and the normal distribution of TDP-43 in the nucleus and cytoplasm. Also, it becomes clear that the abnormal subcellular distribution of TDP-43 closely relates to the pathology by contributing to the formation of insoluble cytoplasmic aggregates.

### Alterations in Liquid-Liquid Phase Separation

Pathological mutations are linked to changes in the LLPS behavior of TDP-43, but the exact pathological mechanisms of the altered LLPS behavior of mutant TDP-43 are still ambiguous. Mutations in the CTD can be divided into two groups. The first group are mutations displaying a direct effect on LLPS and these are located in the helix-loop-helix region in the CTD (Figure 1). This region is a mutational hotspot and importantly, mutations in this region lead to the strongest clinical outcomes (Bolognesi et al., 2019). At a molecular level, these variants distort the intermolecular interactions and destabilize TDP-43 self-interactions, disrupting LLPS and in turn enhancing aggregation (Conicella et al., 2016; Sun and Chakrabartty, 2017). The second group of variants, mutations in the CTD but outside this helix-loop-helix region, are hypothesized to indirectly affect the process of LLPS by impairing the assembly of membraneless organelles due to modified protein-protein interactions. However, the precise mechanism behind this process needs further investigation (Sun and Chakrabartty, 2017).

**FIGURE 3:**
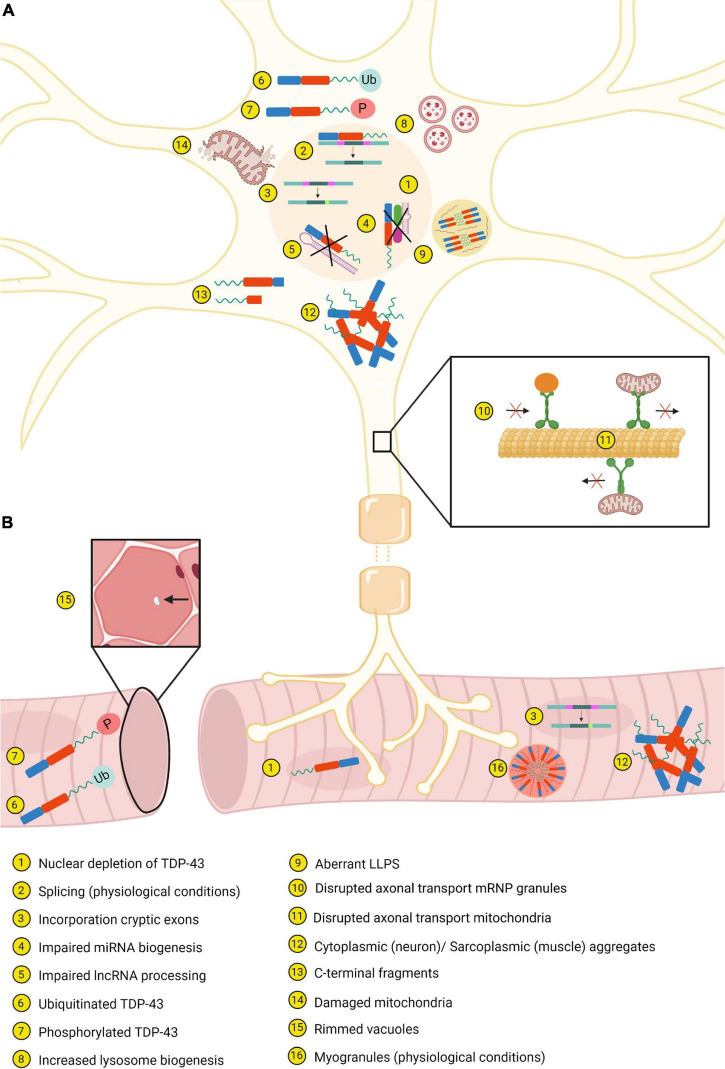
Most important similarities and differences between neurons **(A)** and muscle tissue **(B)**. Created with BioRender.com.

Interestingly the mode of action by which all the CTD variants interfere with LLPS might not only go through a direct effect on the physical process as described above but also indirectly through the induction of alterations in the TDP-43 protein levels and/or its localization. Overexpression of TDP-43 enhances the cytoplasmic TDP-43 levels which disrupt LLPS and finally induces toxicity. This can be explained by an imbalance between TDP-43 and RNA. In the nucleus there is a high concentration of RNA competing with TDP-43 and inhibiting LLPS, whereas in the cytoplasm lower RNA concentrations are present, which are necessary for proper LLPS (Maharana et al., 2018; Mann et al., 2019). In case of excessive TDP-43 in the cytoplasm, there is less RNA to bind all TDP-43 molecules, promoting RNA-deficient TDP-43 interactions, homo-oligomerization of the low complexity domains and subsequently inclusion formation (Mann et al., 2019). This also clarifies how persistent cellular stress, leading to increased levels of TDP-43 in the cytoplasm, contributes to the formation of irreversible pathological aggregates (Sun and Chakrabartty, 2017; Maharana et al., 2018). However, whether the liquid-like TDP-43 foci or the aggregates are the culprits of disease is still under debate. Bolognesi et al. suggest that these aggregates are rather protective, and toxicity is caused by the formation of liquid-like TDP-43 foci clustered at the nuclear periphery (Bolognesi et al., 2019). Basically, failure of LLPS may lead to the formation of fibrillar aggregates and a debate is still ongoing as to what the precise toxicity level is (Figure 3).

As mentioned previously, LLPS is responsible for the formation of several membraneless organelles in both the nucleus and the cytoplasm. When the physical process of LLPS is altered, changes in the functioning and the type of organelles are also an expected outcome. Anisomes are recently identified nuclear membraneless organelles, but the specific function is not described yet. RNA-binding compromised TDP-43, induced by acetylation or disease-causing mutations in the RRMs, forms spherical intranuclear droplets in physiological conditions. These intranuclear droplets are referred to as anisosomes. The shell of anisosomes is densely packed, membraneless and enriched in TDP-43. The center of the droplet contains HSP70 chaperones that interact with the RNA-binding deficient TDP-43 and stabilize it. This indicates that the formation of anisosomes is controlled by the ATP-dependent HSP70 chaperone activity. Treatment of collected rat-derived dorsal root ganglion neurons with a proteasome inhibitor shows the presence of anisosomes when immediately collected. In contrast, in 2-h post-mortem neurons the anisosomes have disappeared and nuclear aggregates formed instead. This indicates that, when ATP-levels are reduced, anisosomes transform into aggregates (Yu et al., 2021). NBs are another type of bodies formed where the RRMs of TDP-43 play a critical role in their assembly. The disease-causing mutation, D169G, in the RRM1 decreases *NEAT1*-RNA binding capacity and therefore reduces the formation of NBs in acute stress conditions. Only with exposure to longer stress conditions is this mutant capable of inducing the assembly of NBs. Remarkably, under these conditions, an increase in the assembly of SGs is seen as compared to WT TDP-43, a process that is not observed during acute stress. The SGs formed during long stress exposure leads toward the formation of phosphorylated cytoplasmic TDP-43 (Wang et al., 2020). In the cytoplasm the SGs are the best-known membraneless organelles involved in the development of a pathological outcome. The ∼25 and ∼35 kDa C-terminal fragments, lacking the NTD and hence the PBMs, are only partially recruited to stress granules by PAR. The failure of these fragments to localize to stress granules gives rise to phosphorylation and aggregation in a cellular system (McGurk et al., 2018).

### Cellular Impairment: Mitochondrial and Autophagic Dysfunction

Mitochondrial dysfunction is observed in patients with ALS and FTD, and these abnormalities have been reflected in several experimental models. *In vivo* and *in vitro* models show that overexpression of disease-associated TDP-43 mutants results in a disruption of both anterograde and retrograde mitochondrial transport in axons (Figure 3; Fazal et al., 2021). Furthermore, the observed mitochondria have an irregular shape, reduced length and accumulate in “grape bunch-like” structures (Wang W. et al., 2013; Magrané et al., 2014; Wang et al., 2016). In addition, for certain TDP-43 mutants, it has been shown that they enhance mitochondrial fragmentation and neuronal death (Figure 3; Wang et al., 2016). Interestingly, TDP-43 binds to mtDNA-derived mRNAs encoding complex I subunits within neuronal mitochondria of ALS and FTD patients and inhibition of this localization blocks neuronal toxicity (Wang W. et al., 2013; Wang et al., 2016). Interestingly, mitochondrial dysfunction is known to augment reactive oxygen species which in turn has been shown to induce TDP-43 aggregation. This might induce a pathological feedback loop, as abnormal TDP-43 may induce mitochondrial impairment which in turn leads to more aggregation of TDP-43 and enforces the cellular toxicity with harmful effects to mitochondria (Prasad et al., 2019).

Impairment of mitochondrial dysfunction is closely related to disrupted autophagy. Damaged mitochondria caused by, e.g., pathological TDP-43, are cleared by autophagy, but functional mitochondria regulate autophagy as well (Huang et al., 2020). Remarkably, TDP-43 is also thought to be crucial in the autophagy-lysosomal pathway (ALP) as loss of TDP-43 function is associated with alterations in the ALP and toxicity. In normal conditions, TDP-43 regulates the mRNA stability of raptor, a component of mTORC1, through binding of the RRMs to the raptor mRNA. However, in case of loss of TDP-43 there is a decrease in the raptor levels and hence increased nuclear translocation of TFEB, an important regulator in the ALP. Consequently, there is an upregulation of genes of the ALP and therefore increased autophagy and lysosome biogenesis (Figure 3). Furthermore, in the absence of TDP-43 the fusion between autophagosomes and lysosomes is disrupted, resulting in the presence of autophagic vesicles (Xia et al., 2016). How pathological TDP-43 further affects the mitochondria and the autophagy, and how these are related to each other are described by Huang et al. (2020).

### Disruption of the RNA Machinery by Loss of Endogenous Function

TDP-43 interconnects with multiple genes crucial for neuronal functioning and development. Depletion of TDP-43 by use of antisense oligonucleotides in mouse brains leads to a downregulation of 239 genes, including *TARDBP* itself. Since a majority of the downregulated genes are important for synaptic activity, loss of TDP-43 could contribute to neuronal disorders (Polymenidou et al., 2011).

Aberrant splicing properties of TDP-43 contribute to the pathology as well. First, in case of loss of nuclear TDP-43 function, there is the incorporation of cryptic exons in several genes (Figure 3). These cryptic exons are commonly observed in neurodegenerative diseases, namely ALS/FTD and AD (Ling et al., 2015; Sun et al., 2017). Second, inhibition of splicing of cryptic intron 6 of *TARDBP* is correlated with increased *TARDBP* mRNA, which in turn leads to increased insoluble and fragmented TDP-43 (Sugai et al., 2019). Finally, D’Alton et al. showed that alternative splicing of *TARDBP* RNA resulted in protein isoforms encoding a unique C-terminal sequence. One of these isoforms directly localized to the cytoplasm and overexpression of the isoform altered the protein levels (D’Alton et al., 2015).

Disruption in the biogenesis of miRNA and processing of lncRNAs has also been described (Figure 3). Depletion of TDP-43 in neuroblastoma cells causes a reduction of neuronal outgrowth. This can be explained by decreased levels of some miRNAs which are normally highly expressed in motor neurons. There is also speculation that cytoplasmic TDP-43 aggregates cannot sufficiently process miRNAs via the Drosha and Dicer complexes (Kawahara and Mieda-Sato, 2012). In contrast, another study suggests that cytoplasmic TDP-43 interacts with miRNAs associated with ALS (Paez-Colasante et al., 2020). Furthermore, in FTD associated with ALS there are increased expression levels of *NEAT1* and *MALAT1*, two lncRNAs which are regulated by TDP-43 (Tollervey et al., 2011).

In mouse cortical neurons and human iPSCs, disease-causing mutations in TDP-43 disrupt the axonal transport of mRNP granules and mitochondria (Figure 3; Chu et al., 2019; Fazal et al., 2021). Interestingly, by studying the interactome of mutant TDP-43, differential interactions with key players in the axonal transport pathway were identified, explaining the observed defects. Moreover, TDP-43 cooperates for its function in transport and translation with other RNA binding proteins. This binding occurs particularly via the CTD of TDP-43. Since the majority of the mutations are located within this CTD, these could act by attenuating the association between TDP-43 and RBPs resulting in impaired transport and translation of mRNA (Alami et al., 2014; Chu et al., 2019; Fazal et al., 2021). Finally, the TDP-43^*A*315*T*^ mutant enhances translation of Dennd4a which in turn increases the expression levels of the *DENND4A* gene, a motor neuron death driver. In mutation-associated ALS cases, it is possible that chronic stress leads to dysregulation of *DENND4A* gene expression, stimulating motor neuron death (Alami et al., 2014).

### Post-translational Modification and the Production of Novel TDP-43 Species

TDP-43 undergoes several post-translational modifications (PTMs) which are associated in both positive and negative ways with TDP-43 pathology. The best studied PTMs are phosphorylation and ubiquitination. Phosphorylation sites on threonine (T), serine (S) and tyrosine (Y) residues, are scattered throughout the entire protein. Hasegawa et al. (2008) suggested that casein kinase 1 or 2 is responsible for the phosphorylation of the most critical residues in the C-terminus. Besides the well-known role of casein kinases, several other kinases have been reported to engage in the phosphorylation of TDP-43 such as cell division cycle kinase 7, glycogen synthase kinase 3, Tau tubulin kinase 1 and 2 (Liachko et al., 2013, 2014; Moujalled et al., 2013). Phosphorylation of six residues in the CTD, S369, S379, S403/404 and S409/410, is frequently found in diseased conditions (Figure 1; Eck et al., 2021). However, especially phosphorylation of S409/410, is suggested as a pathological marker, since phosphorylated S409/410 is only observed in patients with ALS and FTD, and not in healthy controls. For this reason, antibodies against phosphorylated S409/410 are now used as a diagnostic tool for TDP-43 proteinopathy (Inukai et al., 2008). The precise role of phosphorylation of TDP-43 residues in disease is still debated. Disease-causing mutations are found with a dual effect on phosphorylation by either inserting or removing phosphorylation sites (Figure 1; Eck et al., 2021). However, the majority of studies attribute a toxic role to increased phosphorylation status of the protein, thereby triggering the accumulation of oligomers and aggregates with prion-like features able to propagate from cell to cell. Additionally, phosphorylated TDP-43 is associated with loss of physiological functionality, loss of TDP-43 homeostasis and death of spinal motor neurons (Nonaka et al., 2016; Martínez-González et al., 2020). In contrast, some other studies propose that phosphorylation is rather a sign of a protective defense mechanism of the cell, occurring only after aggregation (Brady et al., 2011; Li et al., 2011).

Ubiquitination is another well-studied PTM of TDP-43 and is necessary for the clearance and degradation of soluble full-length TDP-43 via the Ubiquitin proteasome system (UPS). A blockage of this system triggers the formation of insoluble and immobile TDP-43 aggregates and the accumulation of ubiquitylated TDP-43. Full-length TDP-43 aggregates are associated with the presence of K48- and K63-linked ubiquitin chains. Proteins with K48-linked polyubiquitin chains are degraded by UPS-dependent mechanisms, while K63-linked chains target proteins to the autophagic pathway. The fact that full-length TDP-43 aggregates are labeled by both types of ubiquitin chains and inhibition of autophagy prevents the clearance of aggregated TDP-43 indicates an important role for the autophagy pathway in the removal of TDP-43 aggregates in conjunction with UPS-mediated degradation. Impairment of the UPS or autophagy in patients with ALS provokes the accumulation of misfolded and insoluble TDP-43 and prevents the clearance of these aggregates (Scotter et al., 2014). Moreover, aggregation of a disease-causing TDP-43 mutant, M337V, disrupts the UPS in motor neuron-like cells resulting in a depletion of free ubiquitin. This is associated with impairment in neuronal outgrowth and synaptic development (Farrawell et al., 2020). Again, this observation suggests a potential vicious cycle in which mutated TDP-43 leads to UPS dysfunction and further leads to TDP-43 aggregation and UPS dysfunction.

Besides these two common PTMs, other less studied PTMs are linked to pathology as well (Prasad et al., 2019). Acetylation and cysteine oxidation are two of them and both can be triggered by oxidative stress. WT TDP-43 is susceptible to acetylation under physiological conditions, but mislocalized cytoplasmic TDP-43 is more prone. There are 20 Lysine (K) residues in TDP-43, of which only a few are described and characterized in the acetylation process (Prasad et al., 2018). It is speculated that the two major acetylation sites are K145 and K192, both located within the RRMs and resulting in a gain of toxic properties (Figure 1). Directing TDP-43 acetylation-mimics to the cytoplasm results in the formation of phosphorylated aggregates, which colocalize with proteasome and autophagy makers as well as mitochondrial markers (Cohen et al., 2015; Wang et al., 2017). The presence of these markers is suggestive of defects in degradative pathways and mitochondrial impairment (Wang et al., 2017). Besides the presence of the aggregates, the acetylation-mimics have an altered RNA-binding capacity and lose their physiological functions (Cohen et al., 2015). In contrast to the above-described Lysine residues, acetylation of two other Lysine residues in the RRM2, K224 and K263, is linked to a protective role for acetylation (Figure 1). Acetylation of these residues would reduce the *in vitro* aggregation propensity of TDP-43, hypothesized by an increased net charge due to acetylation and hence electrostatic repulsion (Prasad et al., 2018).

Cysteine oxidation of TDP-43 in response to oxidative stress alters solubility and nuclear activity and contributes to TDP-43 pathology. Cysteine residues (C) present in the NLS and the RRMs are all subject to oxidation, however, it is thought that only oxidation of C173 and C175 in RRM1 causes protein aggregation. This oxidation-induced aggregation could be strengthened by oxidation of the residues in the RRM2, C198 and C244 (Figure 1; Chang et al., 2013). Upon oxidation of C173 and C175, together with C198 and C244, a disulfide bridge leads to a reduced RNA binding capacity and impaired RNA splicing concomitant with a higher propensity to form nuclear aggregates. Two disease-causing mutations introducing a novel cysteine residue, G348C and S379C, add strength to the pathological involvement of oxidation as a disease mechanism (Figure 1). Oxidation of these novel cysteine residues caused the production of a dimeric species of ∼90 kDa, indicating that the cysteine-introducing mutations could be pathological by generating abnormally cross-linked TDP-43 species (Cohen et al., 2012).

Another PTM, Poly(ADP-ribosyl)ation (PARylation), is described as modulating the LLPS behavior of TDP-43 through the two PBMs located within the NLS (Figure 1). PARylation is a bidirectional process in which polymers of ADP-ribose (PARs) are added to a subset of amino acids by poly(ADP-ribose) polymerases (PARPs) and can be removed again by enzymes such as PAR glycohydrolase (PARG) (Duan et al., 2019). Based on *in vitro* experiments mutations in the PBMs impair LLPS and thus cause the formation of irregular solid structures (McGurk et al., 2018). In NSC34 cell-lines and *D. melanogaster* models, downregulation of PARP counteracts the toxicity induced by overexpression of WT TDP-43, whereas downregulation of PARG intensifies toxicity. These data indicate the important role of steady-state PARylation as alterations in PARP and PARG can promote toxicity (Duan et al., 2019).

A final PTM of TDP-43 is SUMOylation. SUMOylation is a three-step enzymatic cascade in which small ubiquitin-related modifiers (SUMOs) are conjugated, via an isopeptide bond, with lysine residues in a consensus motif. In TDP-43, K136 and the SUMO-interacting motif (a.a.: 106–110), both located within RRM1, are especially susceptible to this enzymatic process (Figure 1) (Maurel et al., 2020; Maraschi et al., 2021). SUMOylation is thought to modify the functions of TDP-43 as SUMO2/3 colocalizes with the highly expressed nuclear fractions of WT TDP-43 and with the nuclear inclusions of mutated TDP-43 (Seyfried et al., 2010). The precise role of SUMOylation of TDP-43 is not well known but it is suggested to be important for physiological functions such as splicing and nucleocytoplasmic trafficking. Alterations to this enzymatic process in pathological conditions could contribute to TDP-43 pathology by enhancing aggregate formation but require further investigation (Maurel et al., 2020; Maraschi et al., 2021).

### C-terminal Fragmentation Leading to the Production of Toxic TDP-43

TDP-43 C-terminal fragments (CTFs) of ∼25 and ∼35 kDa are prominent species observed in human *post-mortem* tissues derived from ALS patients (Figure 3). Several caspases are involved in the formation of these CTFs. Li et al. suggest that ∼25 kDa CTFs are formed by caspase-4 cleavage, which initiates and activates caspase-3/7. Caspase-3/7 is required for cleavage of TDP-43 into ∼35 kDa fragments, further fragmentation and eventually degradation by stimulating the UPS and autophagy pathway. This cleavage process is necessary for the clearance of full-length TDP-43; however, a small proportion of the cleaved fragments becomes insoluble in pathological conditions and is detected in TDP-43 aggregates. The CTF positive aggregates, mainly processed in the endoplasmic reticulum, will recruit full-length TDP-43 to form additional aggregates and spread them to other locations (Li et al., 2015).

Overexpression of the ∼25 and ∼35 kDa fragments in HEK293T cell-lines indicates that the ∼25 kDa fragments are more susceptible to form numerous and large aggregates, mainly located in the cytoplasm (Zhang et al., 2009; Brady et al., 2011). Moreover, the ∼25 kDa fragments are sensitive to phosphorylation at the S409/410 sites and have increased insolubility when compared to the ∼35 kDa species and full-length TDP-43 (Zhang et al., 2009; Brady et al., 2011). Since the ∼25 kDa fragments have increased phosphorylation and a stronger propensity to aggregate, Brady et al. assume that the level of phosphorylation is related to the degree of aggregation (Brady et al., 2011). Although there is increased phosphorylation of the ∼25 kDa CTF, this phosphorylation is not necessary for its aggregation into inclusions. Together these data suggest that cleavage is a prerequisite for TDP-43 phosphorylation, aggregation and toxicity (Zhang et al., 2009).

### Dimerization and Oligomerization

The same residues in the NTD important for physiological dimerization and oligomerization contribute to TDP-43 pathology as well. In pathological conditions, the first 10 residues are capable of sequestering nuclear full-length TDP-43 into cytoplasmic inclusions and therefore trigger aggregation. This implies that the first residues have both LOF and GOF capacities (Zhang et al., 2013). Along the same line, TDP-43 lacking the NTD produces fewer oligomers but enhance aggregation (French et al., 2019). The idea for protective functions of physiological oligomerization is further supported by Afroz et al. who suggest that due to the N-terminal interactions the low complexity domains in the CTD of adjacent molecules within these oligomers are distant from each other and therefore prevent aggregation. Furthermore, it is speculated that the oligomerization protects against proteolytic cleavage and the formation of toxic C-terminal fragments, a typical hallmark in ALS (Figure 2; Afroz et al., 2017).

Finally, the NTD, together with RRM1, are able to form an ∼86 kDa dimeric form of TDP-43 through intermolecular interactions. Overexpression of this dimer in HEK293T cells triggers the production of aggregates and therefore may be involved in seeding the formation of pathological aggregates (Shiina et al., 2010). Altogether, this indicates that a regular NTD might act as a double-edged sword.

### Aggregation

Aggregation is one of the most typical hallmarks of TDP-43 proteinopathies. The aggregate formation can be triggered by many routes, some already discussed in the previous sections like post-translation modifications, the formation of aberrant TDP-43 species, oligomerization, aberrant LLPS and dysfunction of several cellular processes (Figure 2; French et al., 2019; Prasad et al., 2019). Typically, the aggregates observed in TDP-43 proteinopathy contain, besides WT TDP-43 and modified species of TDP-43, a small proportion of CTFs (Zhang et al., 2009; Li et al., 2015). It is thought that these cleavage products have a physiological function as they are required for the clearance of full-length TDP-43, and hence, protein homeostasis. The ∼25 and ∼35 kDa fragment production, however, comes with a risk as they are very susceptible for aggregation, especially when excessively produced (Li et al., 2015). Besides the fact that the CTD is cleaved into fragments more prone to aggregation, the whole CTD is key in the process of aggregation as it harbors the structural features increasing the propensity for aggregation. The CTD resembles a PLD, known to have a high propensity to aggregate, as it is rich in glycine and uncharged polar amino acids. A complete deletion of this domain reduces the number of aggregates, pointing to the involvement of this domain in the aggregate formation (French et al., 2019). In addition, the CTD has a high probability to fold into ß-sheets. A region in the CTD between a.a. 311–360 presents as a helix-loop-helix structure in solution, but has the tendency to transform into a ß-sheet and as a consequence assemble into aggregates (Jiang et al., 2013). In addition to this region, numerous other regions of the CTD are described to fold into ß-sheets (Chien et al., 2021). Most of the disease-causing mutations are located within the CTD and increase the tendency of TDP-43 to accumulate into aggregates (Johnson et al., 2009; Prasad et al., 2019). In aggregation assays, disease-causing mutations A315T and M337V, located in the CTD, reduce the number of monomers in an early stage and therefore enhance the formation of aggregates (French et al., 2019).

Interestingly hyperexcitability causes the formation of TDP-43 isoforms lacking the CTD. These isoforms are located within the cytoplasm where they aggregate and sequester nuclear TDP-43. Besides the formation of aggregates, these isoforms lack their physiological function such as autoregulation and splicing (Weskamp et al., 2020).

However, neither the CTD nor the NTD by itself harbors the full ability to induce the formation of aggregates. The RRMs are important in the formation of these oligomers and subsequently aggregates as shown by TDP-43 constructs in which the RRMs are replaced by monomeric EGFP. These synthetic proteins display a higher ratio of monomer versus oligomer over time, showing the synergistic function of the RRMs on the aggregation induced by the CTD and NTD (French et al., 2019).

Based on the above data, it appears that alterations to the protein structure sensitize the protein to form insoluble structures.

### Prion-Like Behavior

The CTD of TDP-43 is also referred to as PLD as it contains a segment enriched in glutamine and asparagine residues. This region is used to regulate the self-interaction, aggregation and amyloidogenic propensities of TDP-43 (Smethurst et al., 2016). In prion-like disease, misfolded prion-proteins recruit normal proteins and hence propagate from cell-to-cell (Smethurst et al., 2015, 2016). This process of so-called “seeding” induces the formation of more pathological aggregates and is observed in TDP-43 proteinopathy (Smethurst et al., 2016). Arguments for such a seeding behavior of TDP-43 are increasing of late. Phosphorylated aggregates derived from ALS brains co-transfected with WT TDP-43 into HEK293T cell-lines or neuroblastoma cell-lines show an increase in phosphorylated TDP-43 in comparison with controls, indicating that pathological TDP-43 aggregates are able to seed further aggregation. Cells expressing phosphorylated TDP-43 inclusions co-cultured with pre-labeled acceptor cells shows cell-to-cell propagation, suggesting seeding behavior (Nonaka et al., 2013; Smethurst et al., 2016). Several other *in vitro* and *in vivo* studies have already been described and are reviewed by Jo et al. (2020). Furthermore, the expression of dimeric species in HEK293 cells increases the accumulation of TDP-43 species with a high molecular mass, indicating that this dimer may seed the production of aggregates (Shiina et al., 2010). There are some disease-causing mutations reported that have increased aggregation propensity and enhanced seeding capacity. This indicates that increased aggregation is associated with enhanced cellular seeding activity (French et al., 2019). How the aggregates are propagated from cell-to-cell is still unknown. Some studies assume involvement of vesicles, especially microvesicles and exosomes, in the seeding behavior, however, these are not detected in the study of Nonaka et al. (2013), Feiler et al. (2015), Smethurst et al. (2016), and Jo et al. (2020). Despite the fact that there is still some contradiction as to whether or not vesicles are involved in the cell-to-cell transmission of TDP-43, TDP-43 proteins packed in vesicles are more efficiently taken up by naive HEK293 cells when compared to either free TDP-43 in the culture medium or control empty vesicles (Feiler et al., 2015). Finally, a recent breakthrough study used high resolution cryo-EM analysis of motor and frontal cortices-derived amyloid fibrils from ALS/FTD patients to describe a double spiral structure of pathological TDP-43 filaments formed by residues 282-360 of the low-complexity PLD. This finding is consistent with the hypothesis of prion-like accumulation and propagation of TDP-43 aggregates (Arseni et al., 2021).

## Toward a Muscle-Specific Role for TDP-43

### TDP-43 Inclusions Are Consistently Found in Myopathies

Most of the physiological and pathophysiological processes of TDP-43 are studied in the context of neurodegenerative diseases. Interestingly, strong evidence for a pathological role of TDP-43 in skeletal muscle is provided by the observation that TDP-43 aggregates, reminiscent of the aggregates in neurons, accumulate in patients with sporadic inclusion body myositis (sIBM), IBM associated with Paget’s disease of bone and frontotemporal dementia (IBMPFB), oculopharyngeal muscular dystrophy (OPMD), distal myopathies with rimmed vacuoles (DMRV) and myofibrillar myopathies, including desminopathy and myotilinopathy (Weihl et al., 2008; Küsters et al., 2009; Olivé et al., 2009; Vogler et al., 2018).

Sporadic inclusion body myositis is a progressive myopathy associated with muscular atrophy, especially in the quadriceps and volar forearm muscles, eventually leading to wheelchair dependence. It usually affects people older than 50 years of age and is slightly more commonly observed in males. The disease progression is very slow and therefore it often takes 5–10 years between the onset of disease and the diagnosis (Küsters et al., 2009; Britson et al., 2021). The most prominent features of sIBM are changes with the invasion of CD8 + T-cells, mitochondrial abnormalities, protein aggregates and rimmed vacuoles. It is speculated that sIBM is related to neurodegenerative diseases as the protein aggregates contain amyloid-β, tau and TDP-43, typical hallmarks of ALS, FTD and AD. The presence of TDP-43 positive aggregates is suggestive for the involvement of TDP-43 in the pathology of sIBM, but genetic evidence is lacking. Accumulation of these aggregates causes endoplasmic reticulum stress and calcium dysregulation which in turn results in myofiber degeneration (Huntley et al., 2019; Britson et al., 2021). The idea for the role of TDP-43 in sIBM is further supported by colocalization of pathological TDP-43 and mitochondria in the skeletal muscle of patients. This indicates that mitochondria-associated TDP-43 could contribute to muscle dysfunction, however, further research is necessary to understand the underlying pathological mechanisms (Huntley et al., 2019). Besides sIBM, there is a “quadriceps-sparing” hereditary form of IBM (hIBM) caused by mutations in the *GNE* gene. This hereditary form is also known as DMRV. The first clinical manifestation is an alteration in gait patterns and at a later stage the patients become wheelchair dependent. This disease affects younger patients when compared to sIBM, as the average disease onset is 26 years of age. The muscular pathological hallmarks are akin to those observed in sIBM, but a few differences are noted. First, in DMRV the tibialis anterior muscle is the most affected, while the quadriceps muscles are spared. Second, in DMRV there is no lymphocytic inflammation and there are no mitochondrial-disease markers present. The rimmed vacuoles stand as a common muscle pathological finding between sIBM and DMRV (Askanas and Engel, 1998; Nishino et al., 2005). These rimmed vacuoles are present in other muscular diseases as well, such as OPMD. In OPMD, a typically autosomal dominant progressive myopathy caused by repeat expansions in *PABPN1*, patients develop eyelid ptosis and dysphagia. Usually, these symptoms start to appear in patients between the age of 50–60 years and at a later stage of the disease all voluntary muscles may become affected. In a histopathological context the most prominent hallmark is the presence of intranuclear inclusions. Furthermore, there is muscle atrophy, variation in the size of fibers, enhanced number of nuclei, increased fatty connective tissue and rimmed vacuoles. The rimmed vacuoles present in sIBM, DMRV and OPMD are strongly associated with autopaghy (Brais, 2011).

IBM can be accompanied by Paget’s disease of bone and FTD, referred to as IBMPFB or MSP, and is usually caused by *VCP* mutations. Typically, these patients present with two or more of the diseases together. IBM and FTD are already described and are characterized by muscle weakness and personality/behavioral alterations respectively (Kimonis et al., 2008). In Paget’s disease there is abnormal and enhanced remodeling of the bone composition, which results in softening of the bones and subsequently leads to deformities, pain and pathological fractures (Kimonis et al., 2008; Ralston and Layfield, 2012).

Myofibrillar myopathies represent a group of muscular disorders with variable clinical manifestations depending on the subtype (defined by the causative genes), however, muscle weakness is generally present. Typically, in all myofibrillar myopathies, myofiber degeneration starts around the Z-disc of the sarcomere (Selcen and Engel, 2011). Furthermore, the degraded myofibrillar products accumulate into aggregates and there is ectopic expression of several proteins (De Bleecker et al., 1996; Selcen and Engel, 2011). The subtypes in which TDP-43 aggregates are observed are desminopathy, myotilinopathy and distal hereditary motor neuropathy combined with myofibrillar myopathy, caused by mutations in the *DES*, *MYOT* and *HSPB8* genes, respectively (Selcen and Engel, 2011; Cortese et al., 2018).

### TDP-43 Proteinopathy in Muscle: Toxic Gain-of-Function or Loss-of-Function?

The presence of TDP-43 aggregates in the above-described myopathies strongly supports a broader pathogenic and primary role of TDP-43 in muscle degeneration. Similar to the physiological processes, the pathological processes related to TDP-43 pathology are described in cell-lines and mostly interpreted and confirmed in a neuronal context. In diseased conditions, TDP-43 typically accumulates in the cytoplasm and this accumulation is accompanied by a nuclear depletion. This situation makes the understanding of the pathogenic mechanism complicated as the cytoplasmic accumulation, and hence the aggregation, point toward a gain-of-toxic-function, but in contrast the nuclear depletion may indicate a loss-of-function (Vanden Broeck et al., 2014). This ongoing debate has mainly been focused on a neurological context, but now can be extended to a muscular context as well and will be further discussed.

Evidence for loss-of-function is provided by knock-down and overexpression experiments in *D. melanogaster*. *TBPH* knock-down during muscle development, larval stage and adulthood cause age-related motor abnormalities. These abnormalities involve alterations in the overall motor activity, mean speed and the total distance travelled (Diaper et al., 2013; Strah et al., 2020). *TBPH* knock-down during muscle development and adulthood also affects the lifespan. In addition, *TBPH*-depleted muscles show reduced levels of the Dlg, membrane disorganization and abnormal structure of the presynaptic terminals. The morphological changes can be explained by a decline in the expression levels of *futsch*, the presynaptic microtubule binding protein (Strah et al., 2020). Moreover, *D. melanogaster* overexpressing *TBPH* in the muscles exhibit pre-pupal lethality and impaired motor behavior. The muscle tissues obtained from these larval *D. melanogaster* models show a nuclear depletion of *TBPH* and sarcoplasmic aggregates in muscle fibers. On top of that, the size of the muscle fibers is reduced and the actin filament staining is altered, suggesting that the formation of the sarcomeres is altered during muscle development. This study assumes that, as in neurons, an autoregulatory feedback loop whereby sarcoplasmic aggregates result in nuclear depletion and muscle specific LOF (Diaper et al., 2013).

Additional evidence supporting a LOF mechanism is the observation of increased cryptic exon incorporation in neurons of ALS patients. Interestingly, cryptic exon inclusion has also been observed in sIBM, indicating that TDP-43 is not able to exert its proper normal function (Figure 3). It has been shown that the loss of functional properties and the rimmed vacuoles in sIBM were not induced by cytotoxic T-cells, indicating that the rimmed vacuole muscle degeneration may be associated with the loss of TDP-43 (Britson et al., 2021).

In contrast, cell and animal-based experiments provide a role for a toxic-gain of function of TDP-43. Overexpression of TDP-43 in C2C12 cells, a mouse myoblast cell-line, reduces cell viability and subsequent exposure to acidic conditions further decreases viability and triggers the formation of aggregates. In transgenic mouse models, overexpression of human TDP-43 predominantly in muscles results in ∼35 kDa fragments, increased serum levels of myogenic enzymes (CK, LDH, and AST) and abnormal myofibers. The mean size of the myofibers is smaller than that in the non-transgenic mice, especially in the aggregate-containing myofibers. In addition, the inclusions are enriched in proteins required for protein homeostasis or the UPS pathway and the muscle fibers have autophagic and mitochondrial abnormalities. These data indicate that the aggregates induced by overexpression are not completely able to induce all the pathological aspects of sIBM, since the infiltration of inflammatory cells is lacking. The ∼35 kDa fragments in these transgenic mice may indicate that the ratio of ∼35 kDa over ∼25 kDa fragments is higher in muscle tissues when compared to neuronal tissues (Tawara et al., 2018). Interestingly, a study speculates a tissue-specific difference in the handling of the CTF. The ∼25 kDa CTFs are less sensitive to aggregation in muscle compared to neurons. This difference may be explained by the higher degradative power of the muscle cells. This can be confirmed by proteasomal inhibition in muscle cells expressing the ∼25 kDa fragment that stimulates the autophagy pathway. In general, this suggests that cooperation between the UPS system and autophagy in muscle cells is more effective compared to neuronal cells (Cicardi et al., 2018).

### Reconciling TDP-43 Gain- and Loss-of-Function in Myopathy: The Myo-Granule and Beyond

Interestingly, several findings have provided evidence for both LOF and GOF mechanisms. In a recent hallmark paper, Vogler et al. present *in vivo* experiments in mice that show that cytoplasmic TDP-43 is increased in injured muscle tissue where it forms amyloid-like assemblies, called myo-granules (Figure 3). These myo-granules were found to be enriched in TDP-43 and sarcomeric mRNAs needed to assemble new sarcomeres during muscle regeneration. In normal conditions the myo-granules are cleared within 10 days following the injury (Figure 3). In diseased conditions, increased myo-granule formation or decreased myo-granule clearance may contribute to the formation of aggregates (Vogler et al., 2018). As in neurons, cytoplasmic accumulations consisting of TDP-43 are found in muscle tissue derived from IBM patients. These cytoplasmic accumulations correlate with nuclear depletion and colocalize with phosphorylated TDP-43, ubiquitin and p62, a marker of autophagy (Figure 3; Küsters et al., 2009; Nogalska et al., 2009; Olivé et al., 2009; Salajegheh et al., 2009). The cytoplasmic accumulations could contribute to the GOF pathways, whereas the nuclear depletion suggests LOF properties.

In diseased muscle tissue only the two best studied PTMs, phosphorylation and ubiquitination, are observed, however, a recent study suggests a role for acetylation as well as acetylation mimic mutants in myofibers recapitulate aspects of the sIBM pathology (Nogalska et al., 2009; Olivé et al., 2009; Wang et al., 2017). In cell experiments, WT TDP-43 is mainly present in the nucleus of myofibers, whereas the acetylation-mimic mutant exists as punctate accumulations in both the nucleus and sarcoplasm. When this mutant is directed into the sarcoplasm there is formation of hyperphosphorylated aggregates that co-localize with autophagy, UPS and mitochondrial markers (COXIV, TOM20, cytochrome C). Prolonged expression of the mutant induces the accumulation of large aggregates, and this might be indicative of a failure of the degradative machinery to clear aggregates. Concordantly, the aggregate-containing myofibers are infiltrated by CD8 + T-cells, a typical response observed in sIBM. Finally, TDP-43 positive necrotic myofibers that harbor rimmed vacuoles are present, indicating that the acetylation-mimic mutant may provoke lysosomal damage (Wang et al., 2017).

Overall, these data highlight the importance of TDP-43 in healthy and diseased muscle cells and hint toward an up until now underappreciated but important role of TDP-43 in myopathies.

## A Muscle-Specific Function for TDP-43: Implications for Amyotrophic Lateral Sclerosis

TDP-43 is mainly studied in a neurogenic context since TDP-43 aggregates are a common feature found in several neurological diseases with the most important ones being ALS and FTD. Over the past few years there has been a growing body of evidence for a crucial role of TDP-43 in muscle tissue as well. First of all, several muscle-specific endogenous functions of TDP-43 are described. Furthermore, the pathological hallmarks noted in ALS and FTD resemble the ones in myopathies with rimmed vacuoles, such as sIBM, IBMPFB, OPMD and DMRV (Weihl et al., 2008; Küsters et al., 2009; Olivé et al., 2009). In both myopathies and neurodegenerative diseases besides normal TDP-43, TDP-43 positive aggregates contain abnormal TDP-43 CTF, phosphorylated and highly ubiquitinated species (Weihl et al., 2008; Küsters et al., 2009; Olivé et al., 2009; Lain et al., 2011; Prasad et al., 2019). In addition, the cytoplasmic accumulations can sequester endogenous TDP-43 resulting in nuclear depletion (Olivé et al., 2009). Although TDP-43 aggregates are frequently present in myopathies with rimmed vacuoles, their morphological appearance can differ depending on the patient and type of myopathy. In some patients only a single inclusion is observed, whereas in other patients, multiple aggregates occur (Küsters et al., 2009). Moreover, while the aggregates in sIBM are usually small and punctate, and sometimes, but not always, colocalize with ubiquitin, the aggregates in IBMPFB are large and always colocalize with ubiquitin (Weihl et al., 2008). This is in accordance with FTD in which several types of TDP-43 inclusions are described, as well as microvacuoles (Younes and Miller, 2020). Based on the different morphological appearance and the presence of microvacuoles, it seems that the histopathology of myopathies with rimmed vacuoles is more closely related to FTD than ALS. In general, the different disease entities share a misregulation of endogenous TDP-43 and inclusion formation, pointing toward a broadened disease spectrum ranging from neurodegeneration to myopathy.

This idea is further supported by the surprising presence of phosphorylated TDP-43 in skeletal muscles of patients with ALS, in which muscular atrophy is generally assumed to have a neurogenic origin due to the loss of motor neurons and denervation (Cykowski et al., 2018). However, only a limited number of studies describe the presence of phosphorylated TDP-43 aggregates in ALS muscle and the fraction of ALS patients with muscular TDP-43 aggregates is around 30% (Cykowski et al., 2018; Mori et al., 2019). Moreover, there appears to be a difference in vulnerability according to the type of muscle tissue since phosphorylated TDP-43 is more commonly visualized in axial muscles than appendicular muscles. Further research is needed to see whether muscle abnormalities might contribute to pathogenesis in neurodegenerative diseases as well, however, there is some evidence for cell-autonomous pathology of TDP-43 in the ALS muscle tissue for different reasons. First of all, there is no correlation in severity between the phosphorylated TDP-43 aggregates in the ALS muscles and in the central nervous system (Cykowski et al., 2018). Furthermore, in ALS patients the skeletal and myocardial fibers contain atrophic muscle fibers which have TDP-43 inclusions. The atrophy of the myocardium cannot be attributed to a neurogenic process and therefore the presence of aggregates might indicate the existence of an unexpected primary myogenic disease mechanism (Mori et al., 2019). Finally, TDP-43 overexpression in myoblasts and muscle tissue of mice is toxic on its own as overexpression reduces cell viability, the size of the myofibers and induces the formation of TDP-43 containing inclusions (Tawara et al., 2018). These findings point toward an independent muscle-specific pathological mechanism of TDP-43 aggregates in muscle cells.

## Discussion

TDP-43 is a complex protein with pleiotropic functions in the RNA machinery and is important for the survival of cells and hence the viability of an organism. Steady-state conditions are required for TDP-43 to exert its activity. It is therefore obvious that alterations to TDP-43 homeostasis lead to an increased likelihood of pathological outcomes. TDP-43 is a widely studied protein, but the majority of physiological and pathophysiological functions are mainly studied in the context of neurodegeneration. This is due to the fact that TDP-43 aggregates are common in several neurological diseases, with the most important ones being ALS and FTD. Over the past few years there has been growing evidence for a primary role of TDP-43 in muscle tissue as well, as pathological TDP-43 aggregates are found in muscle tissues from patients with myopathies (sIBM, IBMPFB, OPMD, DMRV and myofibrillar myopathies) and ALS patients alike. This hints toward a need to broaden the disease spectrum of TDP-43 proteinopathy to include, not only neurodegenerative diseases, but also myopathies, further highlighting the need for further research of TDP-43 in a myogenic context.

## Author Contributions

BaD coordinated the manuscript and approved the final version of the manuscript. LV and EB wrote the initial draft. PE, NS, BoD, JD, and BaD critically reviewed and edited the manuscript. LV created the figures. All authors contributed to the article and approved the submitted version.

## Conflict of Interest

The authors declare that the research was conducted in the absence of any commercial or financial relationships that could be construed as a potential conflict of interest.

## Publisher’s Note

All claims expressed in this article are solely those of the authors and do not necessarily represent those of their affiliated organizations, or those of the publisher, the editors and the reviewers. Any product that may be evaluated in this article, or claim that may be made by its manufacturer, is not guaranteed or endorsed by the publisher.
